# Precision neuromodulation: Promises and challenges of spinal stimulation for multi-modal rehabilitation

**DOI:** 10.3389/fresc.2023.1135593

**Published:** 2023-04-19

**Authors:** Maria F. Bandres, Jefferson L. Gomes, Gerson N. Moreno Romero, Avery R. Twyman, Jacob Graves McPherson

**Affiliations:** ^1^ Department of Biomedical Engineering, Washington University in St. Louis, St. Louis, MO, United States; ^2^ Program in Physical Therapy, Washington University School of Medicine in St. Louis, St. Louis, MO, United States; ^3^ Department of Anesthesiology, Washington University School of Medicine in St. Louis, St. Louis, MO, United States; ^4^ Washington University Pain Center, Washington University School of Medicine in St. Louis, St. Louis, MO, United States; ^5^ Washington University Program in Neurosciences, Washington University in St. Louis, St. Louis, MO, United States

**Keywords:** spinal cord injury, neuromodualtion, rehabiliatation, spinal stimulation, locomotion, pain, multimodal rehabilitation

## Abstract

Spinal cord injury results in multiple, simultaneous sensorimotor deficits. These include, but are not limited to, full or partial paralysis of muscles below the lesion, muscle spasms, spasticity, and neuropathic pain. Bowel, bladder, and sexual dysfunction are also prevalent. Yet, the majority of emerging spinal stimulation-based therapies focus on a single issue: locomotor rehabilitation. Despite the enormous potential of these translational advances to transform the lives of people living with spinal cord injury, meaningful recovery in other domains deemed critical priorities remains lacking. Here, we highlight the importance of considering the diverse patterns of neural transmission that underlie clinically similar presentations when developing spinal stimulation-based therapies. We also motivate advancement of multi-modal rehabilitation paradigms, which leverage the dense interconnectivity of sensorimotor spinal networks and the unique ability of electrical stimulation to modulate these networks to facilitate and guide simultaneous rehabilitation across domains.

## Introduction

The sensorimotor consequences of spinal cord injuries (SCI) are wide ranging and intensely individual (1). Unfortunately, the rehabilitation and spinal stimulation fields have yet to adequately address this inherent heterogeneity, despite an increasing focus on precision medicine initiatives in medicine more broadly. Although SCI presents unique challenges within a precision medicine framework, the ability of the central nervous system to reorganize and repair also presents unique possibilities for these approaches.

Recent and emerging technological advances in spinal electrical stimulation systems have positioned the neurorehabilitation field to capitalize on so-called “smart”, or person-specific, neuromodulatory therapies. But the full potential of precision neuromodulation is unlikely to be realized without a dedicated focus on certain key areas of research. Here, we motivate the neurorehabilitation and neural engineering fields to consider some of the basic scientific and translational areas we feel are critical for strategic growth of this exciting new application of spinal stimulation.

We begin by revealing the diversity of intraspinal neural transmission in a ubiquitous pre-clinical model of SCI, highlighting the pitfalls of implicitly assuming homogeneity across animals (or people) when developing neuromodulatory therapies to enhance rehabilitation. We then focus on the promise of closed-loop spinal electrical stimulation paradigms, including the importance of therapies that promote beneficial neural plasticity and afford multi-modal therapeutic benefits. And finally, we discuss the need for increasing our understanding of network-level sensorimotor dynamics, both in the neurologically intact spinal cord and following SCI.

## SCI results in diverse sensorimotor consequences not matched by the scope of neuromodulatory therapy development

It is well documented that impaired voluntary motor control is a canonical feature of SCI, owing to reduced neural transmission in motor pathways descending from the brain to regions of the spinal cord below the lesion. In contrast, spinal responses to sensory feedback often become pathologically increased below the lesion, resulting in hyperreflexia. Overactive spinal responses to sensory feedback also contribute to SCI-related neuropathic pain (SCI-NP) in ∼40%–70% of people living with incomplete SCI (2, 3). Physical disabilities associated with SCI are exacerbated by the presence of SCI-NP, which can limit or even prevent participation in physical therapy and dramatically reduce overall activity level. In addition to the psychosocial burden this places on people living with SCI, the consequences of limited activity for the SCI population are severe. Pressure ulcers, cardiovascular decline, and metabolic changes are all leading causes of morbidity after SCI (1) that are exacerbated by limited activity.

Beyond a general understanding that each individual's clinical sequela is somehow related to their unique injury profile, few neural mechanisms have been identified that consistently predict a person's symptom severity, the extent to which they will respond to a given therapy, or their overall recovery potential. As a result, there is often a paucity of actionable mechanistic information with which to guide therapy. This lack of knowledge has far too often led to a one-size-fits-all approach to interventions and a high degree of variability in therapeutic efficacy across individuals.

**Figure 1:**
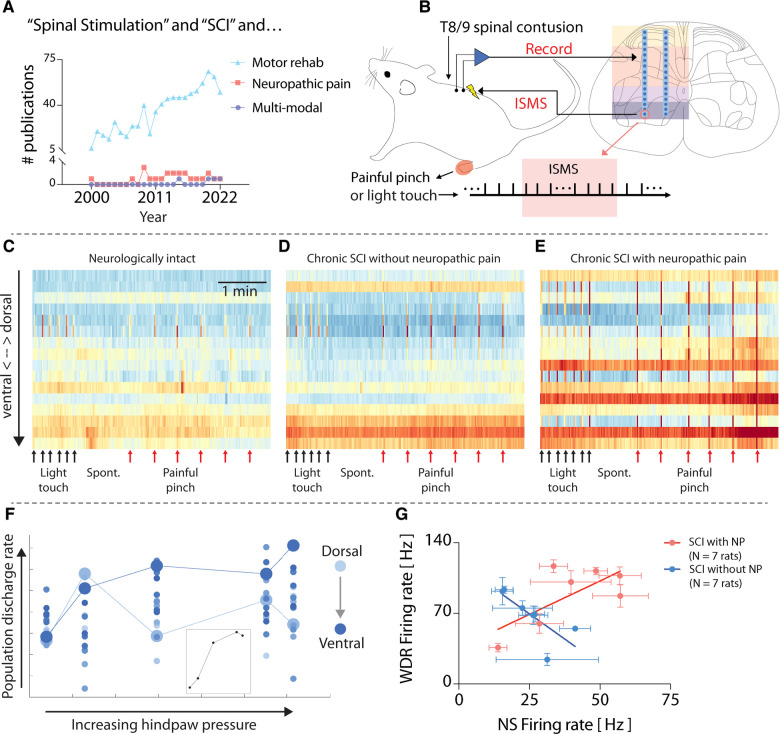
Heterogeneity in spinal responses to natural sensory feedback following spinal cord injury. (**A**) Results of a National Library of Medicine/PubMed database search for manuscripts related to spinal stimulation for sensorimotor consequences of SCI. Search terms: “((spinal cord injury[Title/Abstract]) AND (electrical spinal stimulation)) NOT (functional electrical stimulation[Title/Abstract]) AND (motor)”; “((spinal cord injury[Title/Abstract]) AND (Electrical spinal stimulation)) AND (neuropathic pain)”; “((spinal cord injury[Title/Abstract]) AND (electrical spinal stimulation)) AND (multimodal).” (**B**) Schematic overview of methods used for original data and figures reported in this manuscript. Microelectrode arrays are 32-channel A2 × 16 probes (NeuroNexus), implanted into the lumbar enlargement at the L5 dorsal root entry zone. ISMS is delivered at ∼90% resting motor threshold. Adapted from (4). (**C–E**) Time-resolved histograms of multi-unit spike counts across one shank of the microelectrode array implanted into each rat. Warmer colors indicate more spikes per unit time; cooler colors indicate fewer spikes. Each panel depicts representative data from a single animal. (**F**) Discharge rate of multi-unit spiking activity across one shank of the electrode array implanted into a rat with chronic thoracic SCI in response to punctate pressure applied to the plantar surface of the hindpaw (Von Frey test). The color gradient indicates the depth of each electrode channel, with lighter colors being more dorsal and deeper colors being more ventral. Enlarged circles (light and dark blue) highlight paradoxical behavior in sensory- and motor-dominant regions (respectively) of the spinal cord. (**G**) Firing rate of functionally classified wide dynamic range (WDR) and nociceptive specific (NS) neurons across 14 rats with similar motor impairments following chronic thoracic SCI. The plot reveals the presence of distinct subgroups of rats based on the presence (*N* = 7) or absence (*N* = 7) of SCI-related neuropathic pain (SCI-NP). All animal data presented in this figure was collected as part of protocols approved by the Institutional Animal Care and Usage Committee at Washington University in St. Louis.

Given the diverse sensorimotor consequences of SCI, it is reasonable to wonder how the research portfolio of the neuromodulation field compares. A recent search of the National Library of Medicine PubMed database returned 820 manuscripts since 2000 describing spinal stimulation-based approaches for enhancing motor recovery after SCI (Figure 1A). By comparison, a search of the same timeframe returns merely 23 manuscripts detailing spinal stimulation-based approaches for SCI-related neuropathic pain, and only 4 manuscripts explicitly seeking to develop multimodal spinal stimulation-based approaches for SCI rehabilitation (Figure 1A). Thus, the field could clearly do more to ensure that its research investments accurately reflect the needs of the population it serves.

## Differences in clinical efficacy may be related to underlying neural dynamics

What could contribute to the differences in clinical efficacy of neuromodulatory therapies across people living with SCI? Pre-clinical models can offer clues. Using dense microelectrode arrays implanted into the gray matter of the spinal cord *in vivo* (Figure 1B), it is possible to reveal patterns of neural transmission spanning sensory- and motor-dominant networks. This preparation has allowed characterization of “spontaneous” neural transmission (i.e., that occurring in the absence of sensory feedback), neural transmission during periods of natural sensory feedback, and during electrical spinal stimulation (5, 6). For example, Figure 1C depicts intraspinal neural transmission in a neurologically intact rat (i.e., without SCI), while Figures 1D,E depict rats with chronic motor incomplete SCI (>6 weeks post-SCI). Both rats in Figures 1D,E received an identical moderate-to-severe thoracic contusion injury, and both spontaneously recovered a similar degree of voluntary hindlimb movement. However, the rat in Figure 1D showed no signs of SCI-related sensory abnormalities, whereas the rat depicted in Figure 1E developed behavioral signs of SCI-NP. Despite their similar motor impairments, differences in network behavior are clearly apparent. In particular, unexpected and dramatic differences are evident in the motor-dominant ventral horns of the two animals during induced nociceptive transmission.

Such underlying differences are often masked when considering only population-level analyses, however. This phenomenon is illustrated in Figure 1F. Here, the neural population discharge rate, separated by electrode depth in the gray matter (color intensity gradient), is plotted vs. the magnitude of punctate pressure applied to a receptive field on the plantar surface of the hindpaw in a rat with chronic thoracic SCI. The inset in Figure 1F depicts the population discharge rate averaged across all electrode depths, which predictably increases monotonically, albeit non-linearly, until saturation.

But this trend belies the differences evident across individual regions in the spinal cord. Two responses are highlighted. The first, indicated by the enlarged light blue circles, depicts the discharge rate of a population of neurons in a region of the dorsal horn associated with nociceptive transmission. The second, indicated by the enlarged dark blue circles, depicts the discharge rate of a population of neurons in a region of the ventral horn most closely associated with motor-related transmission. Paradoxically, it is the motor-dominant region that shows the most direct association with increasing pressure.

To further illustrate the underlying differences between animals with effectively the same degree of motor impairment severity, Figure 1G depicts the firing rate of wide dynamic range (WDR) vs. nociceptive specific (NS) neurons in 15 animals with chronic SCI. Here again, the population level trend, which shows a direct, albeit modest, relationship, belies two easily separable subgroups: animals without SCI-NP and those exhibiting behavioral signs of SCI-NP. The indirect relationship between WDR firing rate and NS firing rate in rats without SCI-NP could be taken as consistent with a non-sensitized spinal cord, wherein a gate theory-like phenomenon (7) remains intact and low threshold sensory transmission (which increases WDR firing rate) is associated with a coincident decrease in NS firing rate. In animals with SCI-NP, however, the direct relationship between WDR and NS neurons could be taken as evidence of a state of central sensitization and a breakdown of low-threshold inhibition of nociceptive neural transmission. Because spinal stimulation for motor rehabilitation, whether delivered *via* skin surface electrodes, epidural electrodes, or intraspinal electrodes, recruits motoneurons *via* polysynaptic reflex pathways nominally involving low-threshold afferents and interneurons, the differences in network activity illustrated in Figure 1G suggest that motor-targeted spinal stimulation will act *via* different, if overlapping pathways. These differences may also have implications for therapeutic efficacy.

**Figure 2:**
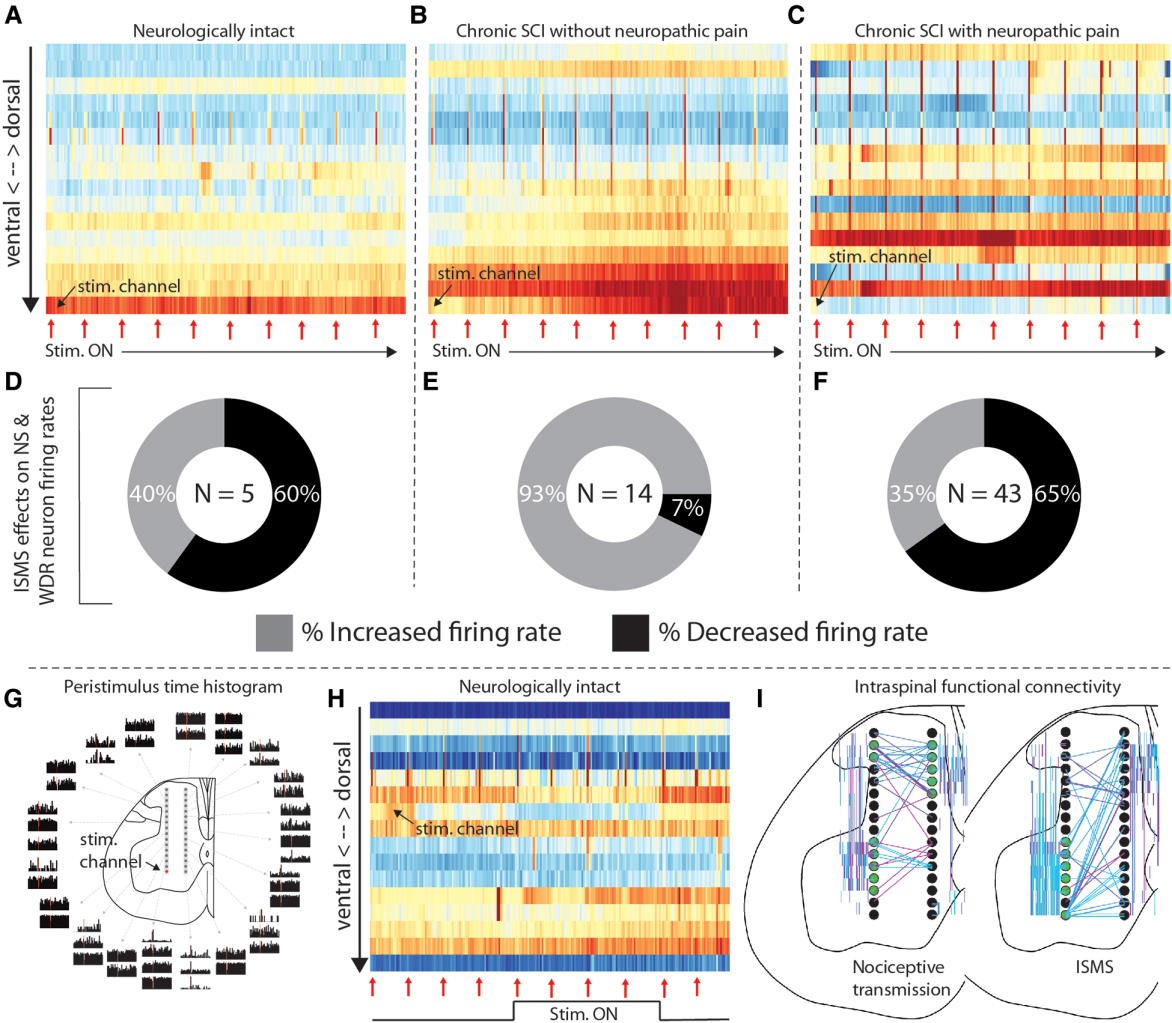
Intraspinal microstimulation for sensorimotor SCI rehabilitation. (**A–C**) Time-resolved histograms of multi-unit spike counts across one shank of the microelectrode array implanted into each rat during sub-motor threshold ISMS (rats and electrodes are the same as those in Figures 1C–E). Warmer colors indicate more spikes per unit time; cooler colors indicate fewer spikes. Red arrows beneath the plots indicate the times that painful pressure was applied to the plantar surface of the hindpaw. (**D–F**) Proportion of functionally classified WDR and NS neurons exhibiting firing rate increases or decreases during motor-targeted ISMS, corresponding to panels **A–C**. (**G**) Peristimulus time histograms of single-unit spiking activity associated with sub-motor threshold ISMS of the lateral-ventral most electrode of the array. Adapted from (4). (**H**) Representative example of the effects of deep dorsal ISMS on neural transmission in sensory- and motor-dominant regions of the spinal gray matter in a single animal. Red arrows beneath the plots indicate the times that painful pressure was applied to the plantar surface of the hindpaw. (**I**) Representative examples of intraspinal functional connectivity during induced nociceptive transmission (left) and motor-targeted ISMS (right). Black circles are schematic representations of the microelectrode arrays. Electrodes highlighted in green are the most connected nodes of the network. Colored lines indicate single neurons statistically determined to be functionally connected, as in (5); warmer colors indicate increasing connection strength. All animal data presented in this figure was collected as part of protocols approved by the Institutional Animal Care and Usage Committee at Washington University in St. Louis.

And indeed, motor-targeted intraspinal microstimulation (ISMS) differentially modulates neural transmission across these animals. Figures 2A–C depicts an epoch of 5 min of sub-motor threshold ISMS delivered to the ventral horn of each of the rats in Figures 1C–E. Although neural transmission is generally enhanced in the intermediate gray matter and ventral horns in all rats, the specific patterns vary widely. And interestingly, although ISMS was parameterized to enhance motor output, it also differentially modulated spinal responses to induced nociceptive transmission across the animals.

Figures 2D–F depicts the proportion of neurons classified as NS or WDR that either increased (lighter color) or decreased (darker color) its firing rate when painful peripheral stimuli were episodically delivered during the period of motor-targeted ISMS shown above (red arrows in Figures 2A–C). The majority of NS and WDR neurons in the neurologically intact animal and the animal with SCI-NP (Figure 2D—60%, and Figure 2F—65%, respectively) exhibit depressed responses to induced nociceptive neural transmission within only the first 5 min of ISMS. In contrast, only 7% of NS and WDR neurons exhibited depressed responses to induced nociceptive transmission in the animal with chronic SCI lacking behavioral signs of SCI-NP (Figure 2E). This observation raises numerous questions from a translational standpoint, many of which center about how to optimize the delivery of spinal stimulation for the specific behavioral and network-level presentation of each animal.

## Discussion

In the face of such profound heterogeneity, and with the knowledge that the clinical efficacy of currently available spinal stimulation paradigms is not broadly generalizable, what strategies can the neurorehabilitation and neural engineering fields employ to address the unmet need for precision neuromodulation? Of the myriad possibilities, the following three interrelated areas seem ripe for consideration.

### Closed-loop neuromodulation

First, the field could more readily focus on closed-loop neuromodulation. In closed-loop paradigms, delivery of spinal electrical stimulation is contingent upon and synchronous with detection of a functionally relevant neural event. The goal of this approach is to enhance the intrinsic ability of the central nervous system to reorganize and repair by restoring and reinforcing natural patterns of neural transmission in spinal networks below the lesion. It is distinguished from open-loop stimulation, in which the delivery of stimuli is not correlated with a specific neural event. For example, ISMS of motor pools associated with forelimb extension can be synchronized with the detection of volitional attempts to recruit those muscles, evinced from motor unit action potentials in the target muscles (8). This approach leads to significantly greater recovery of fine motor control in rats with chronic cervical SCI than does open-loop stimulation, wherein stimulation of the same motor pools is uncorrelated with volitional intent. Presumably, the greater efficacy of closed-loop stimulation is related to induction of functionally relevant neural plasticity in weakened yet spared spinal networks. And indeed, closed-loop stimulation leads to therapeutic gains that persist for weeks after stimulation is discontinued, further distinguishing it from open loop paradigms in which the majority of therapeutic benefits require maintained delivery of stimulation (8). As a result, closed-loop paradigms open the door to neuromodulatory therapies intended to *rehabilitate* lost function rather than *replace* lost function.

Biomechanical events can also trigger spinal stimulation in a closed-loop paradigm. Reduced yet detectable movements of the ankle, knee, and hip have been used to trigger epidural stimulation of spinal segments associated with producing those movements for locomotion (9, 10). The extent to which biomechanically triggered epidural spinal stimulation leads to functionally meaningful therapeutic benefits after discontinuation of stimulation has not been rigorously investigated. However, it stands to reason that this approach would still be more effective at driving durable neuroplastic changes than open-loop epidural stimulation, particularly when paired with physical rehabilitation. Regardless, because closed-loop paradigms use an individual's own neural or biomechanical cues to trigger stimulation, they are intrinsically “smart” and patient-specific. As such, this general approach holds considerable promise for overcoming the limitations of the one-size-fits-all paradigms that have been explored to date.

Optimization of closed-loop spinal stimulation paradigms is not trivial, however. Indeed, even small amounts of electrical current delivered directly to spinal motor pools can lead to widespread modulation of neural transmission over a region far exceeding that which would be predicted from direct current spread alone (4, 11). This phenomenon is illustrated in Figure 2G [adapted from (4)], wherein single pulses of sub-motor threshold ISMS were delivered to the ventral horn (red dot; lateral-ventral most electrode on array) while simultaneously recording extracellular neural activity throughout the dorsal horn, intermediate gray matter, and ventral horn. Peristimulus time histograms of the neural activity are shown according to location in the gray matter. The theoretical region of direct current activation is approximately the same size as the schematic illustration of the electrode on which it was delivered (i.e., the red dot). Modulation of neural activity beyond the sphere of direct activation is clearly evident, raising intriguing yet complex questions such as (a) what is an appropriate stimulus trigger for closed-loop stimulation and (b) how is closed-loop ISMS so effective at driving neural plasticity considering its rather surprising lack of spatiotemporal specificity? Insights gained from a deeper understanding of this broad distribution of modulatory effects may also offer clues for how to optimize epidural and transcutaneous spinal stimulation paradigms to enhance their capacity for driving neural plasticity.

### Multi-modal rehabilitation

Spinal stimulation paradigms currently available clinically, as well as those currently undergoing clinical testing, are parameterized for one consequence of SCI alone. Overwhelmingly, reports of new spinal stimulation paradigms and technologies focus on enhancement of locomotion (although SCI-related bladder function continues to be a highly productive area of research). Yet, as aforementioned, a multitude of sensorimotor consequences affect people living with SCI. Although locomotion is routinely rated amongst the top rehabilitation priorities of people living with paraplegia following SCI, it is rarely ranked as a greater priority than bowel, bladder, or sexual function (12).

Amelioration of pain is frequently noted as a priority for individuals living with SCI-NP. And although spinal stimulation paradigms for management of non-SCI-related chronic pain are clinically available, they have yet to gain traction for SCI-NP. Interestingly, though, it has recently been demonstrated in pre-clinical models that ISMS parameterized to enhance voluntary motor output concurrently reduces nociceptive neural transmission integral to the development and persistence of SCI-NP (4). However, direct stimulation of the deep dorsal horn—a region of the spinal gray matter closely associated with pain-related neural transmission—may be more efficacious for management of SCI-NP than motor-targeted stimulation of the ventral horn, while still preserving the ability to enhance voluntary motor output *via* recruitment of sensorimotor reflex pathways. The potential for such an effect is illustrated in Figure 2H, in which reduced neural transmission in sensory networks is accompanied by increased transmission in motor networks during deep dorsal horn ISMS. Although much additional study will be required to optimize this approach for multi-modal benefits, these are nevertheless exciting early demonstrations that such dual effects could be possible.

A key challenge both for pain-related (specifically) and multi-modal (broadly) applications is determining how to configure such systems for closed-loop operation. Finding the optimal trigger sources for stimulation will be essential. For systems parameterized only to enhance voluntary motor output, these sources are somewhat straightforward. But for a phenomenon as enigmatic and multi-faceted as pain, considerably more basic scientific discovery is warranted. It is encouraging to see that clinical research in this area is beginning to emerge for non-SCI-related applications (13), as presumably these efforts will help to guide future pre-clinical and clinical studies for SCI-NP.

As a final note on the development of multi-modal rehabilitation platforms, we strongly encourage researchers and clinicians already pursuing epidural spinal stimulation paradigms—especially those intended to enhance motor output—to incorporate into their studies assessments of off-target effects including sensation and pain. Because ISMS requires additional time to move through the translational pipeline, determining the extent to which epidural spinal stimulation could also serve as a viable candidate for providing multi-modal therapeutic benefits would be an invaluable resource. Given the wealth of pre-clinical and clinical work already ongoing in the epidural spinal stimulation space and the existence of many efficient, validated instruments for assessing sensation and pain, this would seem to be a straightforward way to meaningfully advance the field.

### Spinal network analyses

Finally, an essential step in moving towards smart, more efficacious, spinal stimulation paradigms is elucidating how patterns of intraspinal neural transmission relate (or not) to action and perception. As highlighted throughout Figures 1, 2, traditional approaches, which have either been reductionist (e.g., single- or multi-unit firing rates) or high-level (e.g., motor impairment severity), have not yielded the combination of granularity and stability required to generalize across the SCI population. This is particularly true for SCI-NP. One challenge has been that many approaches assume stationarity in neuron or network firing dynamics, which is rarely borne out by experimental data; rather, spatiotemporal transience appears to be a hallmark of spinal sensorimotor transmission [for example, see (14)]. But a more formidable challenge is that the appropriate substrate or level of analysis required to ascertain a more robust signature of function is not readily evident. This complicates our understanding of the extent to which the patterns can be generalized from one person to another or whether generalization would even be necessary if a more sensitive marker was available.

We suggest that a more detailed characterization of network-level patterns of intraspinal neural transmission associated with movement, pain, or other functions is a tractable yet physiologically rich entry point. There is much to be gained in this area by adapting to the spinal cord the concept of functional connectivity, now well-established in supraspinal networks. For example, it has recently been reported that seemingly spontaneous discharge patterns and associated intraspinal functional connectivity may be non-random and physiologically purposeful (albeit far from fully understood) (5, 6). In Figure 2I, we highlight examples of different connectivity patterns during induced nociceptive transmission and, separately, motor-targeted ISMS. Eventually, network-level signatures such as these could serve as a type of “biomarker”, revealing natural, adaptive patterns of neural transmission that should be reinforced and maladaptive patterns that should be minimized or replaced. They could also aid contextualization of off-target effects, guide selection of stimulation parameters to maximize the opportunities for multi-modal benefits, and be used to track the impact of therapy on recovery.

It is indeed an exciting time in the neuromodulation field, with the pace of technological development and translation seemingly accelerating daily. But along with this rapid pace comes the need to renew our focus both on the diverse, multi-faceted priorities of people living with SCI and on our conceptualization of the spinal cord as a dynamic, integrated system wherein no single function can be modulated in isolation. With these considerations in mind, the promise of precision neuromodulation and multi-modal rehabilitation may become a reality sooner rather than later.

## Data Availability

The raw data supporting the conclusions of this article will be made available by the authors, without undue reservation.
